# Improving inclusion and exclusion criteria in foodborne illness outbreak investigations: a case study

**DOI:** 10.1017/S0950268820000138

**Published:** 2020-02-07

**Authors:** M. J. Firestone, P. Lee, C. W. Hedberg

**Affiliations:** Division of Environmental Health Sciences, University of Minnesota School of Public Health, Minneapolis, USA

**Keywords:** Epidemiology, food-borne infections, outbreaks, salmonellosis

## Abstract

The practice of foodborne illness outbreak investigations has evolved, shifting away from large-scale community case-control studies towards more focused case exposure assessments and sub-cluster investigations to identify contaminated food sources. Criteria to include or exclude cases are established to increase the efficiency of epidemiological analyses and traceback activities, but these criteria can also affect the investigator's ability to implicate a suspected food vehicle. A 2010 outbreak of *Salmonella* ser. Hvittingfoss infections associated with a chain of quick-service restaurants (Chain A) provided a useful case study on the impact of exclusion criteria on the ability to identify a food vehicle. In the original investigation, a case-control study of restaurant-associated cases and well meal companions was conducted at the ingredient level to identify a suspected food vehicle; however, 21% of cases and 22% of well meal companions were excluded for eating at Chain A restaurants more than once during the outbreak. The objective of this study was to explore how this decision affected the results of the outbreak investigation.

## Introduction

In 2017, there were 841 foodborne illness outbreaks reported in the United States and Puerto Rico, resulting in 14 481 illnesses, 827 hospitalisations, 20 deaths and 14 food product recalls [1]. While outbreak cases represent a small subset of all foodborne illnesses [2], outbreak investigations are useful to stop further illness transmission and to identify opportunities for prevention. Since these investigations are iterative processes, typically conducted in field settings when transmission may be ongoing, the investigative approaches and selection of analytic techniques may be limited by practical considerations and available control measures.

Three types of evidence are used to evaluate sources of contamination in foodborne illness: epidemiological evidence, environmental health evidence, including traceback investigations and evidence from laboratory testing [3]. Case-control studies are a useful epidemiological tool during a foodborne illness investigation to generate and test hypotheses regarding risk factors among cases [4]. The practice of foodborne illness outbreak investigations has evolved, shifting away from large-scale community case-control studies to more focused case exposure assessments and sub-cluster investigations to identify contaminated food sources [5, 6]. These epidemiological methods include comparisons of cases and well meal companions rather than community controls, restricting analyses to exposures specific to the sub-cluster.

Criteria to include or exclude cases are established to increase the efficiency of epidemiological analyses and traceback activities, but these criteria can affect the investigator's ability to implicate a suspected food vehicle. A 2010 outbreak of *Salmonella enterica* ser. Hvittingfoss infections associated with a quick service restaurant chain (Chain A) provided a useful case study to assess the impact of exclusion criteria on the ability to identify a food vehicle. The objective of this study was to examine how exclusion criteria affected the results of the epidemiological investigation.

On 27 May 2010, the Illinois Department of Public Health (IDPH) began to investigate an outbreak of *S.* Hvittingfoss infections associated with multiple Chain A restaurants in multiple counties that occurred between April and June 2010. There were 97 patron cases and 12 food handlers with confirmed infections, with illness onsets ranging from 25 April to 30 June [7]. On 2 June 2010, Chain A restaurant locations in the distribution area where the outbreak was occurring were directed to pull four suspected produce items (onions, lettuce, tomatoes and green peppers) based on an early review of the individual food items most frequently consumed by cases. Early in the investigation, green peppers were strongly suspected based on interviews with cases and well meal companions, as well as product traceback data. However, for the final analysis, cases and well meal companions who ate at Chain A multiple times during the outbreak were excluded because it could not be determined which of the meal dates led to the actual exposure. In the final analysis, green peppers were not statistically associated with illness, while lettuce, olives and tomatoes were. Thus, green peppers were not implicated as the food vehicle.

## Methods

To examine how the exclusion of cases and well meal companions with multiple eating dates impacted the findings from epidemiological analyses, de-identified outbreak records were obtained from the IDPH via a freedom of information act (FOIA) request. Data were aggregated and analysed to reproduce results summarised in the report. There were 85 cases and 32 well meal companions. The IDPH excluded 18 cases and 7 well meal companions with multiple eating dates, which represented the exclusion of 21% of cases and 22% of well companions with interview information. The ultimate IDPH case-control analysis included 67 cases and 25 well meal companions. For some cases only positive food exposures were recorded (e.g., some data were entered as 1 = yes if the food was consumed, but all other values were missing). To adjust for this a dataset was created that converted missing values to 0 = not consumed.

Univariate analyses including patrons with multiple meal dates were conducted, and odds ratios (ORs) and 95% confidence intervals (CI) were calculated. We included onions in univariate analyses since they were removed from restaurants during the outbreak along with green peppers, tomatoes and lettuce. These ingredients were reported in the original study because they were consumed by more than one-third of cases [7]. While analyses were conducted for all ingredients, results are only presented for the ingredients that were reported in the original IDPH investigation. Multivariable analyses of statistically significant (at *P*-value <0.05) food items for patrons with single eating dates and multiple eating dates were calculated. Since olives are not a fresh produce item and *Salmonella enterica* is unlikely to survive on olives [8], we conducted multivariable analyses with only green peppers, lettuce and tomatoes. The mean number of fresh produce items that were consumed by patrons and standard errors (s.e.) was also calculated for cases and well meal companions and a *t*-test was conducted to compare these values. Statistical analyses were conducted using Stata version 14.2 (StataCorp LLC, College Station, TX).

## Results

Table 1 shows the results of the univariate case-control analysis excluding patrons who had eaten at Chain A more than once during the outbreak. Three food items were statistically associated with illness – lettuce, tomatoes and olives. In a univariate analysis of all patrons, including those with multiple Chain A meals, green peppers were also significantly associated with illness (OR 3.6; 95% CI 1.2–10.2); however, the magnitude of the association was largest for olives (OR 6.2; 95% CI 1.7–22.5) and tomatoes (OR 3.8; 95% CI 1.4–9.8) (Table 2). All of the excluded patrons who consumed green peppers were cases (*n* = 10). Onions were not statistically associated with illness in univariate analysis excluding those with multiple meal dates (OR 1.4; 95% CI 0.5–3.6) or when including those with multiple meal dates (OR 2.0; 95% CI 0.9–4.8). Most of the patrons with missing values were cases. Including missing values as zeros (which assumes that missing values equated to no consumption) markedly reduced the magnitude of association for olives (OR 4.0; 95% CI 1.1–14.4) and tomatoes (OR 2.6; 95% CI 1.1–6.4) (Table 3). Green peppers were the only food item for which these transformations increased the odds of exposure among cases.

**Table 1.** Case-control study for *S.* Hvittingfoss outbreak (cases and well meal companions with single meal dates), Illinois, April – June 2010

	Case	Well-meal companion	OR	95% CI	*P*-Value
Green peppers					
Ate	21	5	2.1	(0.7, 6.4)	0.19
Did not eat	40	20			
% Consumed	34.4%	20.0%			
Lettuce					
Ate	56	15	4.1	(1.4, 12.0)	0.01*
Did not eat	9	10			
% Consumed	86.2%	60.0%			
Tomatoes					
Ate	54	16	3.4	(1.1, 9.9)	0.03*
Did not eat	9	9			
% Consumed	85.7%	64.0%			
Olives					
Ate	20	2	7.9	(1.7, 37.5)	0.01*
Did not eat	29	23			
% Consumed	40.8%	8.0%			
Onions					
Ate	27	10	1.4	(0.5, 3.6)	0.49
Did not eat	29	15			
% Consumed	48.2%	40.0%			

* *p* <0.05.

**Table 2.** Case-control study for *S.* Hvittingfoss outbreak (cases and well meal companions, including those with multiple eating events), Illinois, April – June 2010

	Case	Well-meal companion	OR	95% CI	*P*-Value
Green peppers					
Ate	31	5	3.6	(1.2, 10.2)	0.02*
Did not eat	47	27			
% Consumed	39.7%	15.6%			
Lettuce					
Ate	68	19	3.3	(1.3, 8.3)	0.01*
Did not eat	14	13			
% Consumed	82.9%	59.4%			
Tomatoes					
Ate	69	20	3.8	(1.4, 9.8)	0.01*
Did not eat	11	12			
% Consumed	86.3%	62.5%			
Olives					
Ate	25	3	6.2	(1.7, 22.5)	0.01*
Did not eat	39	29			
% Consumed	39.1%	9.4%			
Onions					
Ate	37	11	2.0	(0.9, 4.8)	0.11
Did not eat	35	21			
% Consumed	51.4%	34.4%			

*p < 0.05

**Table 3.** Case-control study for *S.* Hvittingfoss outbreak (cases and well meal companions, including those with multiple eating events), Illinois, April – June 2010 (missing values assumed to equate to no consumption)

	Case	Well-meal companion	OR	95% CI	*P*-Value
Green peppers					
Ate	31	5	3.1	(1.1–8.9)	0.04*
Did not eat	54	27			
% Consumed	36.5%	15.6%			
Lettuce					
Ate	68	19	2.7	(1.1–6.6)	0.03*
Did not eat	17	13			
% Consumed	80.0%	59.4%			
Tomatoes					
Ate	69	20	2.6	(1.1–6.4)	0.04*
Did not eat	16	12			
% Consumed	81.2%	62.5%			
Olives					
Ate	25	3	4.0	(1.1–14.4)	0.03*
Did not eat	60	29			
% Consumed	29.4%	9.4%			
Onions					
Ate	37	11	1.5	(0.6–3.4)	0.37
Did not eat	48	21			
% Consumed	43.5%	34.4%			

* *p* < 0.05

In multivariable analyses restricted to patrons with single exposures, lettuce had the strongest magnitude of association (OR 2.8, 95% CI 0.9–9.2) although no food items were statistically associated with illness (Table 4). By contrast, when including patrons with multiple meal dates, green peppers had the strongest magnitude of association (OR 2.9, 95% CI 1.0–8.7) (Table 5). This was also true when including missing values (Table 5). Overall, mean consumption of produce items was significantly higher among cases compared to well-meal companions when including patrons with multiple eating events (3.7 *vs.* 2.8 respectively; *P*-value = 0.02).

**Table 4.** Multivariable analysis case-control study for *S.* Hvittingfoss outbreak (cases and well meal companions with single meal dates), Illinois, April – June 2010

	OR	s.e.	95% CI	*P*-Value
Green peppers	1.7	1.0	(0.5–5.6)	0.35
Lettuce	2.8	1.7	(0.9–9.2)	0.08
Tomatoes	1.9	1.2	(0.6–6.4)	0.31

**Table 5.** Multivariable analysis case-control study for *S.* Hvittingfoss outbreak (cases and well meal companions, including those with multiple eating events, missing values as missing and with missing values assumed to equate to no consumption), Illinois, April–June 2010

	Missing values not included				Missing values assumed to equate to no consumption			
	OR	s.e.	Lower CI	*P*-value	OR	s.e.	Lower CI	*P*-value
Green peppers	2.9	1.6	(1.0–8.7)	0.05	2.6	1.4	(0.9–7.6)	0.09
Lettuce	2.1	1.1	(0.8–5.9)	0.14	2.1	1.0	(0.8–5.6)	0.14
Tomatoes	2.2	1.2	(0.8–6.4)	0.15	1.6	0.8	(0.6–4.3)	0.39

## Discussion

In this outbreak, the removal of four produce items (lettuce, tomatoes, green peppers and onions) that had been eaten by at least 36% of cases appeared to successfully stop the transmission of illness. There was only one case with an illness onset date beyond the window of an incubation period after the call for the removal of the four produce items. While the odds ratio for olives was the highest, olives were not one of the food items that was removed from the restaurants, but outbreak transmission was still effectively stopped. The original outbreak investigation did not definitively implicate a single food vehicle, but rather listed lettuce, tomatoes or olives as possible food vehicles because they were shown to be statistically associated with illness [7]. While the ingredient-specific analysis cannot clearly implicate a single food vehicle, including those with multiple meal dates showed that green peppers were associated with illness as was found early in the initial outbreak investigation. This finding could have helped inform the outbreak investigation in real-time and, in conjunction with traceback and/or laboratory evidence, informed a less ambiguous conclusion. Inaccurate or ambiguous findings in outbreak investigations can cause significant financial implications for the food industry. In 2008, the tomato industry suffered considerable losses after tomatoes were mistakenly implicated as the source of a *Salmonella* Saintpaul outbreak [9] highlighting the importance of clear findings in outbreak investigations.

Produce items including tomatoes and lettuce were distributed from a central Illinois distribution centre that served multiple restaurant customers including Chain A. However, green peppers were only delivered to Chain A restaurants. Confirmed patron cases reported eating at 49 Chain A restaurants in 28 Illinois counties. All but three confirmed cases ate at restaurants within the same produce distribution area [7]. Thus, combining the traceback data with the epidemiology strongly suggests that green peppers were the likely vehicle in this outbreak.

A previous review of restaurant inspection results from outbreak and non-outbreak restaurants concluded that Chain A restaurants likely served as a pass through for the contaminated produce to patrons, without amplifying the contamination [10]. Low-level contamination of fresh produce items has been associated with prolonged incubation periods [11]. Thus, relatively low levels of contamination of produce would be consistent with the observed mean incubation period of 4 days (ranges 0 to 15 days) for cases with only one reported meal date.

Epidemiological evidence from foodborne illness outbreak investigations is a critical component of an investigation. Despite being initially suspected, green peppers were not ultimately implicated when restaurant patrons with multiple eating dates were excluded. This study showed that excluding patrons with multiple meal dates from case-companion analyses changed the conclusions that could be drawn from the results of the original investigation. Using all available information to construct a coherent narrative of what happened and why is a critical component of an outbreak investigation.

There are several limitations to this study. First, this was a secondary analysis of these data and there was a limited ability to recreate the original analyses as noted in the IDPH investigation report. In particular, when applying the original exclusion criteria for the re-analysis, there was one additional case who reported eating lettuce. This, however, did not likely impact the findings of the secondary analysis. Furthermore, the original report noted excluding eight well meal companions who had multiple meal dates. However, upon review of the data obtained via FOIA request, one of these companions reported being ill and should not have been excluded for that reason, rather than for reporting multiple meal dates. As a result, only seven additional companions were included in the analysis with patrons who had eaten at Chain A more than once during the outbreak.

Additionally, there were several cases for which data was only entered if the item was consumed and was otherwise left blank. In order to reduce this affirmation bias, we looked at these data as if missing indicated the food item was not consumed. Since we do not have the ability to know if these food items were not consumed or were unintentionally overlooked in the interview, we may have unintentionally introduced misclassification bias in these findings. Given that the data were not systematically entered, however, this bias is likely minimal as missing values likely represented non-consumption. Furthermore, for cases with multiple eating dates, we did not have the ability to distinguish which food items were eaten during each occasion, which limits the ability to show a dose–response relationship at the ingredient level. Finally, the analysis was limited by a small number of controls and collinearity among food items. When including patrons with multiple eating dates, cases consumed significantly more produce items than well meal companions.

Despite these limitations, this study is useful for exploring the impact of exclusion criteria on what conclusions can be drawn from outbreak investigations. All of the excluded cases who consumed green peppers became ill. *Salmonella* infections are dose dependent [12], and multiple eating occasions may represent an increased dose. The average incubation period for cases with multiple meal dates (3.1 ± 0.7) was shorter than those with single eating dates (4.5 ± 0.5). Green peppers were the only item where odds of exposure increased as more data were included in the analyses.

Outbreak investigation practices are shifting with the application of whole genome sequencing (WGS). Realising the benefits of WGS will require improved exposure assessments of sub-clusters to guide food item traceback investigations. Using all available case data will be critical to the success of these investigations. When making decisions about inclusion and exclusion criteria, sensitivity analyses to assess the impact of these decisions will be important for determining whether the impact justifies the action.
